# Analysis of the correlation between EAT thickness and prognosis in patients with heart failure with preserved ejection fraction

**DOI:** 10.3389/fcvm.2025.1640707

**Published:** 2025-09-05

**Authors:** Jianfei Ma, Hongbin Zhang, Ting Ma, Fei Liu

**Affiliations:** ^1^ The Third Department of Cardiovascular, Cangzhou Central Hospital, Cangzhou, Hebei, China; ^2^ The Third Department of Ultrasound, Cangzhou Central Hospital, Cangzhou, Hebei, China

**Keywords:** EAT thickness, heart failure with preserved ejection fraction, prognosis, risk factors, heart failure

## Abstract

**Purpose:**

To explore the correlation between epicardial adipose tissue (EAT) thickness and prognosis in patients with heart failure with preserved ejection fraction.

**Method:**

A total of 156 patients diagnosed with heart failure with preserved ejection fraction (HFpEF) were selected as the observation group. Another 150 healthy persons undergoing physical examination were selected as the control group. According to the 1-year follow-up results of prognosis of HFpEF patients, they were further divided into a good prognosis group (112 cases) and a poor prognosis group (44 cases).

**Result:**

The EAT thickness, left ventricular mass index (LVMI), left atrial diameter (LAD), and left ventricular end-diastolic diameter (LVEDD) of HFpEF patients in the observation group were higher than those of the control group, and their LVEF was lower than that of the control group. EAT thickness was negatively correlated with LVEF, and EAT thickness was positively correlated with LVMI, LAD, and LVEDD. Hemoglobin and estimated renal glomeruli of the poor prognosis group were lower than those of the good prognosis group. The EAT thickness, blood lactate, and serum creatinine in the poor prognosis group were higher than those in the good prognosis group. Reduced hemoglobin, increased EAT thickness, and increased blood lactate were risk factors for poor prognosis in patients with HFpEF. The receiver operating characteristic analysis showed that the optimal EAT cutoff value was 5.65 mm (area under the curve = 0.892, 95% CI = 0.833–0.936), with 89.13% sensitivity and 88.39% specificity. For the Kaplan–Meier survival analysis, when performing the *post hoc* stratification of event-free survival, we applied a clinician-relevant threshold of 7.59 mm.

**Conclusion:**

EAT thickness is inversely related to HFpEF severity and reflects left ventricular remodeling. EAT thickness may be a potentially useful non-invasive prognostic indicator of poorer outcomes in patients with HFpEF.

## 1 Introduction

Heart failure is the terminal stage of the progression of various cardiovascular diseases. The patient's own heart develops systolic or diastolic dysfunction, and it has a high mortality rate. Approximately 40 million heart failure patients are reported worldwide every year, and the mortality rate exceeds 10% (1, 2). Heart failure patients can be divided into three categories according to left ventricular ejection fraction (LVEF): heart failure with reduced ejection fraction, heart failure with preserved ejection fraction (HFpEF), and mildly reduced ejection fraction heart failure (3). HFpEF is a heterogeneous syndrome with multiple comorbidities, multiple cardiac and extracardiac pathophysiological abnormalities, and multiple phenotypic manifestations (4). There is currently no effective treatment for HFpEF, and few treatments can effectively improve patient survival rates. The focus of treatment is only on optimizing risk factors for HFpEF.

HFpEF continues to present challenges for treatment because of its heterogeneity of pathogenesis and lack of effective treatments, making prognostication an important area of research (5).

Epicardial adipose tissue (EAT) is a metabolically active depot of visceral fat located between the myocardium and the visceral pericardium that is in contact with coronary vessels and myocardial tissue, ultimately leading to both local inflammation and cardiac remodeling. EAT enlarges abnormally in patients with HFpEF and may mediate this process by secreting proinflammatory and fibrotic cytokines, leading to impaired relaxation of the myocardial wall and diastolic dysfunction (6, 7).

That said, the prognostic significance and potential to use EAT thickness as a prognostic marker among patients with HFpEF is not well evaluated, particularly among East Asian population groups in which there is a greater prevalence of non-obesity phenotypes ,with overall body composition differing from that of Western profile groups. Thus, existing findings are not transferable in East Asian cohorts, highlighting the need to investigate these region-specific data (8, 9).

For this reason, this study investigated the association of EAT thickness and clinical outcomes in HFpEF patients from an East Asian, non-obese population. Using transthoracic echocardiography to evaluate EAT, we were also able to show that EAT could be examined as a non-invasive biomarker for risk stratification and prognostication (10).

## 2 Materials and methods

### 2.1 General information

A total of 156 patients diagnosed with HFpEF in our hospital from August 2020 to August 2022 were selected as the observation group. Another 150 healthy persons undergoing physical examination during the same period were selected as the control group. This study was approved by the Ethics Committee of Cangzhou Central Hospital. Informed consent was also obtained from all participants.

Inclusion criteria consisted of meeting the diagnostic criteria for HFpEF in the European Society of Cardiology Heart Failure Association consensus “The HFA-PEFF diagnostic algorithm” (11) and the patient being >18 years old. Patients were excluded if they had severe liver and kidney dysfunction; severe arrhythmia, congenital heart disease, pulmonary heart disease, rheumatic valvular heart disease, and other heart diseases; acute cerebrovascular disease, malignant tumors, or infectious diseases; or communication barriers and low compliance.

### 2.2 EAT measurement and echocardiography

**Figure 1:**
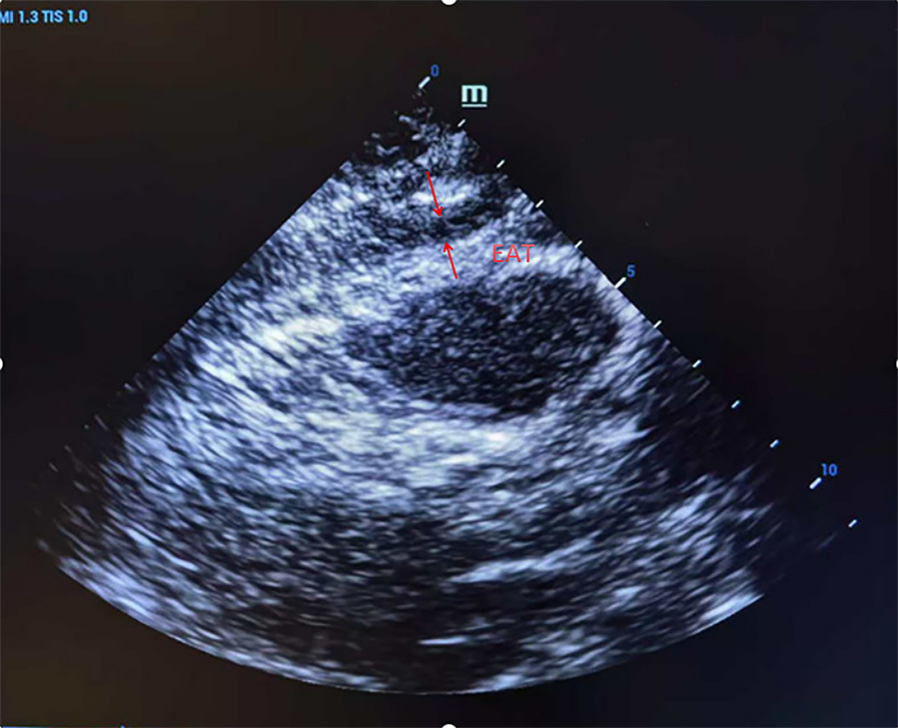
Ultrasound detection of right ventricular anterior epicardial adipose tissue (EAT) thickness in parasternal long-axis view. A representative echocardiographic image showing a measurement of EAT thickness located between the right ventricular free wall and the visceral pericardium, taken at end-diastole.

EAT measurement was assessed with Doppler ultrasonography (Shanghai Longyi Medical Devices Co., Ltd, model: XF3500). Measurement consisted of an initial low-frequency probe (2.5–3.5 MHz), followed by a high-frequency probe (7.5–10 MHz) for better resolution. In the parasternal long-axis view, we drew a line perpendicular to the right ventricular free wall level from the aortic annulus. At end-diastole, we measured fat thickness (EAT) between the free wall of the right ventricle and the visceral pericardium in five consecutive cardiac cycles, and we recorded the mean value (Figure 1).

Echocardiography was performed using a color Doppler echocardiograph (manufacturer: Mirion, model: Econo) with a three-dimensional matrix probe (1.5–3.5 MHz). The parameters that were measured include left ventricular ejection fraction (LVEF), left atrial volume index (LAVI), the ratio of early diastolic mitral inflow velocity to early diastolic mitral annular velocity (*E*/*e*′), and left ventricular mass index (LVMI).

Six experienced cardiologists independently assessed EAT thickness and echocardiographic indices. Intraobserver and interobserver variability were then assessed for 30 randomly selected cases, and the results yielded intraclass correlation coefficients (ICC) of 0.92 and 0.89, respectively. All measurements were performed in a blinded assessment to avoid influencing the patient's prognosis.

### 2.3 Prognostic follow-up and grouping

Medical record number, address, and contact information for each HFpEF patient were collected for long-term follow-up. Patients were followed up through hospital records and telephone contact until 30 November 2023. Endpoint events included readmission for heart failure and all-cause death during the follow-up period. Events were adjudicated by an independent committee of two senior cardiologists.

Poor prognosis was defined as one or more endpoint events occurring within the 1-year follow-up period. A good prognosis was defined as survival without heart failure (HF)-related readmission. Of the 44 patients in the poor prognosis group, 40 were hospitalized and six died. Of the 112 patients in the good prognosisgroup.

### 2.4 Analysis of risk factors for poor prognosis

General clinical data, historical medical data, physical examination data, laboratory values, and medication data were captured. Laboratory measurements were done from fasting peripheral venous blood samples collected on the morning of admission.

When available, B-type natriuretic peptide (BNP) or N-terminal prohormone of B-type natriuretic peptide (NT-proBNP) data were evaluated for ventricular dysfunction. Discharge medication data included the use of angiotensin-converting enzyme inhibitors or angiotensin II receptor antagonists, beta-blockers, and diuretics; additional medication data included the use of guideline-directed therapies such as MRAs, SGLT2, and ARNI.

### 2.5 Statistical analysis

All statistical analyses were performed using SPSS 25.0. A *post hoc* power analysis was performed and indicated that the number of events (*n* = 44) satisfied the ≥10 events-per-variable assumption of multivariable analysis.

Continuous variables were examined for normality with the -Shapiro–Wilk test. Normally distributed variables were expressed as mean ± standard deviation and compared with independent sample *t*-tests. Non-normally distributed variables were analyzed with the -Mann–Whitney *U*-test. Categorical data were expressed as counts and percentages. Categorical data were analyzed with the chi-squared test or Fisher's exact test.

Echocardiographic measures (LVEF, LAVI, LVMI, and *E*/*e*′), comorbidities (hypertension or diabetes), and medication type (antihypertensives, antidiabetics, ASA, and statins) were included in the univariate analyses. Precise values of *P* < 0.10 were included in the multivariable logistic regression model.

Pearson correlation was used to describe the relationship between EAT thickness and echocardiographic indices. A receiver operating characteristic (ROC) curve analysis was performed to determine the predictive value of EAT thickness for adverse outcomes. -A Kaplan–Meier survival analysis with a log-rank test was performed. Calibration of the models was performed using the Hosmer–Lemeshow goodness-of-fit test. Statistical significance was set at a two-sided value of *P* < 0.05.

## 3 Result

### 3.1 Comparison of general information between the two groups of patients

The age of patients in the observation group ranged from 38 to 78, with an average of 56.79 ± 7.04. There were 81 male patients and 75 female patients. The average body mass index was 24. 83 ± 0.62 kg/m^2^. The age of the control group patients ranged from 26 to 77, with an average of 55.6 1 ± 11.32. There were 75 male patient and 75 female patients. The average body mass index was 24.56 ± 4.66 kg/m^2^. No statistically significant difference was found in the general information of the two groups of patients (*P* > 0.05).

### 3.2 Comparison of EAT thickness and echocardiographic indicators

EAT thickness, LVMI, LAVI, and *E*/*e*′ were higher in HFpEF patients in the observation group than in the control group (*P* < 0.05), and LVEF was lower than in the control group (*P* < 0.05). The results are given in Table 1.

**Table 1:** Comparison of EAT thickness and echocardiographic indicators between HFpEF patients and healthy subjects.

Group	EAT thickness (mm)	LVEF (%)	LVMI (g/m^2^)	LAVI (ml/m^2^)	E/e′
Control group	5.10 ± 1.16	62.37 ± 5.01	104.43 ± 18.56	28.76 ± 4.19	7.59 ± 1.36
Observation group	6.80 ± 1.63	59.00 ± 4.31	125.35 ± 20.42	38.18 ± 5.35	13.49 ± 2.86
t	10.453	6.347	9.390	17.104	22.845
*P*	<0.001	<0.001	<0.001	<0.001	<0.001

EAT, epicardial adipose tissue; LVEF, left ventricular ejection fraction; LVMI, left ventricular mass index; LAVI, left atrial volume index; *E*/*e*′ is the ratio of peak early diastolic mitral orifice flow velocity (*E*) to peak early diastolic septal-side and left ventricular lateral wall-side mitral annular mean motion velocity (*e*′).

### 3.3 Correlation analysis between EAT thickness and echocardiographic indicators

**Figure 2:**
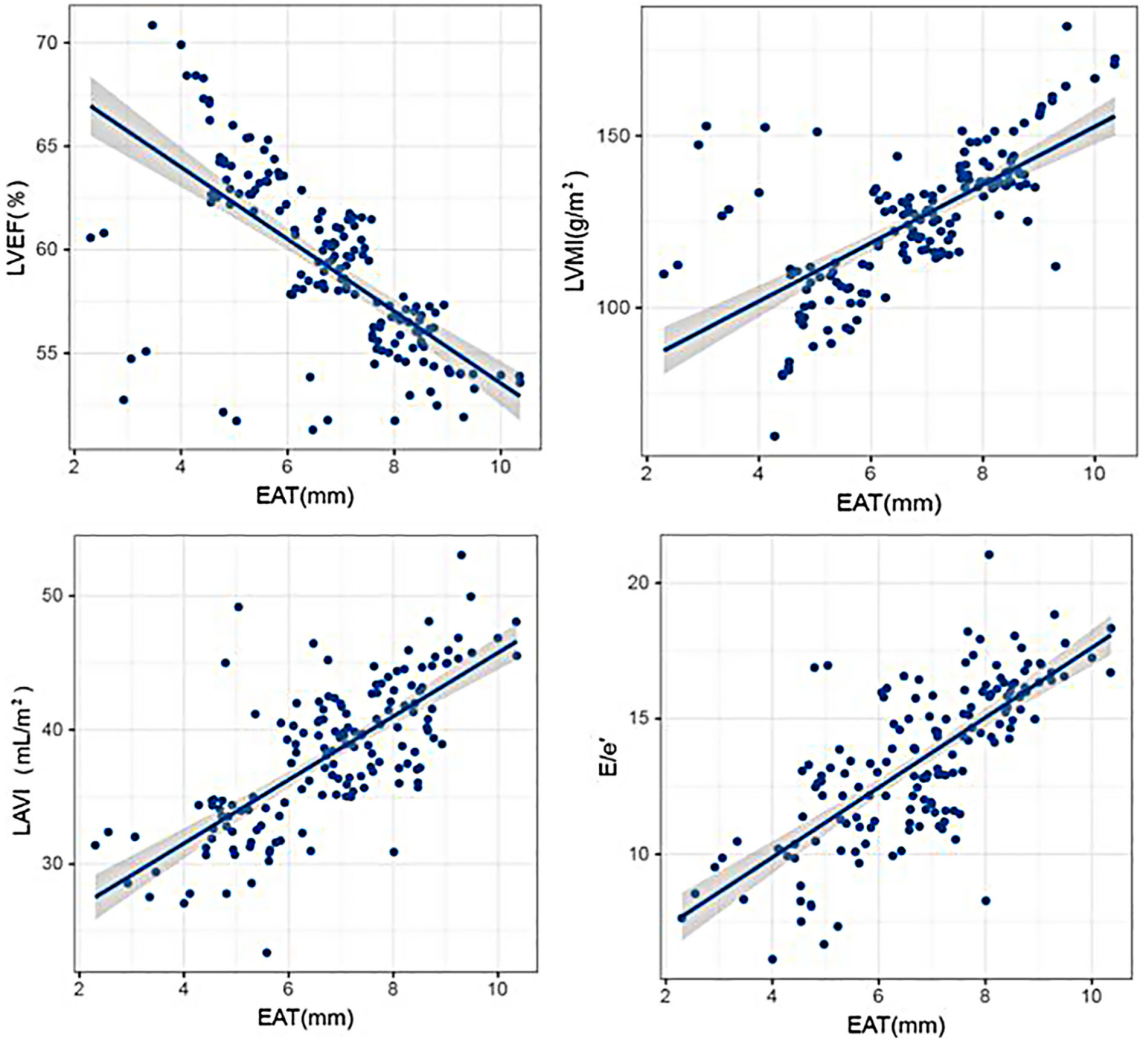
Correlation analysis between EAT thickness and echocardiographic indicators. Scatter plots illustrating the relationships between EAT thickness and key echocardiographic parameters, including LVEF, LVMI, LAVI, and *E*/*e*′, with statistical trend lines indicating significant associations.

Pearson correlation analysis showed that EAT thickness was negatively correlated with LVEF (*P* < 0.05), and EAT thickness was positively correlated with LVMI, LAVI, and *E*/*e*′ (*P* < 0.05). The results are given in Table 2 and Figure 2.

**Table 2:** Correlation analysis between EAT thickness and echocardiographic indicators.

Index	*r*	*P*
LVEF	−0.523	<0.001
LVMI	0.611	<0.001
LAD	0.405	<0.001
LVEDD	0.413	<0.001

EAT, epicardial adipose tissue; LVEF, left ventricular ejection fraction; LVMI, left ventricular mass index; LAD, left atrial diameter; LVEDD, left ventricular end-diastolic diameter.

### 3.4 Single-factor analysis affecting the poor prognosis of HFpEF patients

In this study, the total number of patients with poor prognosis at the end of follow-up was 44. The results of a univariate analysis showed that the clinical data of age, sex ratio, body mass index, smoking history, drinking history, combined hypertension, combined diabetes mellitus, systolic blood pressure, diastolic blood pressure, triglyceride, total cholesterol, low-density lipoprotein, high-density lipoprotein, and medication use in the poor prognosis group had no statistically significant differences when compared with that of the good prognosis group (*P* > 0.05). Hemoglobin and estimated renal glomeruli (eGFR) levels in the poor prognosis group were lower than those in the good prognosis group (*P* < 0.05). EAT thickness, blood lactate level, and serum creatinine (Scr) level were higher in the poor prognosis group than in the good prognosis group (*P* < 0.05). The results are presented in Table 3.

**Table 3:** Single-factor analysis affecting poor prognosis in patients with HFpEF.

Project	Good prognosis group (*n* = 112)	Poor prognosis group (*n* = 44)	*t/X^2^*	*P*
Age years	56.36 ± 7.21	57.89 ± 6.42	1.204	0.231
Gender (e.g., male/female)	56/56	25/21		
Body mass index (kg/m^2^)	24.82 ± 0.65	24.87 ± 0.54	0.466	0.642
Smoking history [cases (%)]	45 (40.18)	11 (23.91)	3.770	0.052
Drinking history [cases (%)]	43 (38.39)	17 (36.96)	0.029	0.866
Combined with hypertension [cases (%)]	20 (17.86)	9 (19.57)	0.063	0.801
Complicated with diabetes [cases (%)]	17 (15.18)	8 (17.39)	0.120	0.729
Systolic blood pressure (mmHg)	136.26 ± 12.06	135.36 ± 11.70	1.483	0.140
Diastolic blood pressure (mmHg)	73.56 ± 4.10	72.13 ± 4.26	1.965	0.057
TG (mmol/L)	1.78 ± 0.32	1.74 ± 0.36	0.606	0.545
TC (mmol/L)	4.62 ± 1.04	4.871.44	1.249	0.213
LDL-C (mmol/L)	2.63 ± 0.57	2.65 ± 0.68	0.233	0.816
HDL-C (mmol/L)	1.01 ± 0.31	0.99 ± 0.22	0.403	0.687
FPG (mmol/L)	5.82 ± 1.54	6.23 ± 1.58	1.507	0.134
Hemoglobin (g/L)	132.56 ± 4.38	121.32 ± 4.10	14.938	<0.001
EAT Thickness (mm)	6.18 ± 1.45	8.28 ± 0.94	9.015	<0.001
Blood lactate (mmol/L)	4.52 ± 0.55	6.04 ± 0.94	12.626	<0.001
Scr (μmol/L)	79.85 ± 18.62	97.07 ± 21.93	5.008	<0.001
eGFR [ml/(min·1.73 m^2^)]	77.40 ± 19.39	65.73 ± 17.69	3.526	<0.001
Medication status [cases (%)]				
Antiplatelet	57 (50.89)	23 (50.00)	0.010	0.919
Loop diuretics	35 (31.25)	14 (30.43)	0.010	0.920
ACEI/ARBs	45 (40.18)	19 (41.30)	0.017	0.896
Beta-blockers	36 (32.14)	14 (30.43)	0.044	0.834

TG, triglyceride; TC, total cholesterol; HDL-C, high-density lipoprotein; LDL-C, low-density lipoprotein; EAT, epicardial adipose tissue; Scr, serum creatinine; eGFR, estimated renal glomeruli. Global filtration rate; ACEI/ARBs are angiotensin-converting enzyme inhibitors/angiotensin II receptor antagonists.

### 3.5 ROC curve analysis of the predictive value of EAT thickness for poor prognosis in patients with HFpEF

**Figure 3:**
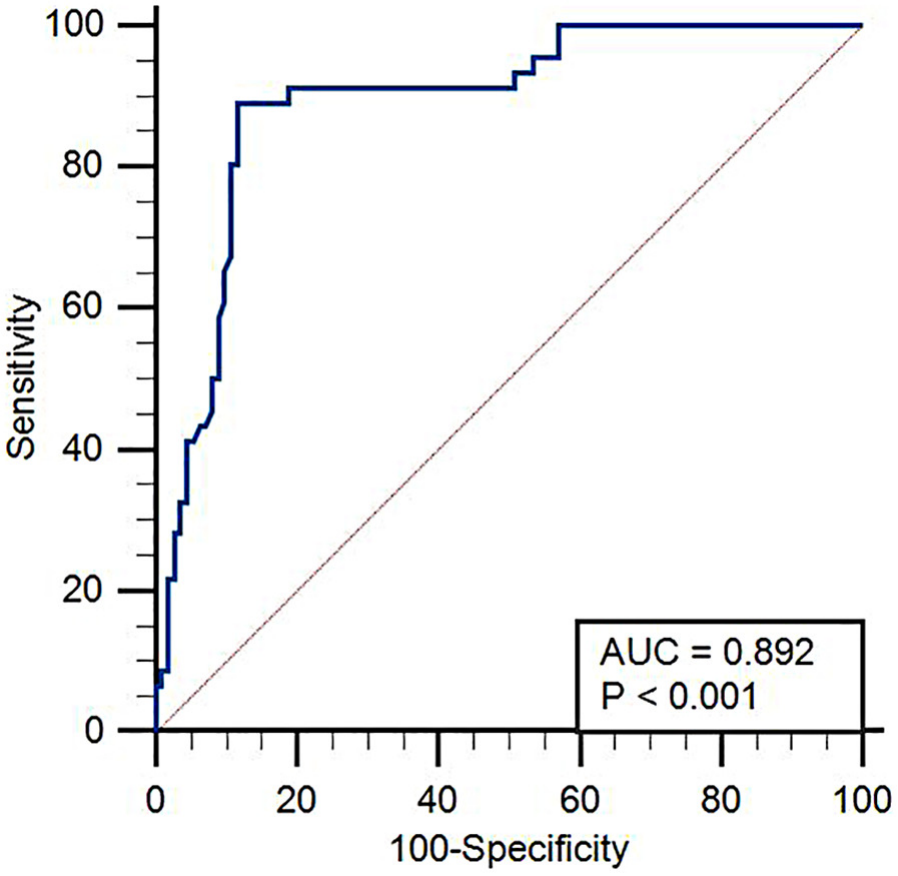
ROC curve analysis of the predictive value of EAT thickness for poor prognosis in HFpEF. Receiver operating characteristic (ROC) curve demonstrating the diagnostic performance of EAT thickness in predicting poor outcomes in patients with HFpEF. The area under the curve (AUC), sensitivity, and specificity values are shown.

The cutoff value of EAT for predicting poor prognosis in HFpEF patients was 5.65 mm, its area under the curve (AUC) was 0.892 (95% CI = 0.833–0.936), and the sensitivity and specificity were 89.13 and 88.39, respectively. The results are shown in Figure 3.

### 3.6 Kaplan–Meier survival analysis of HFpEF patients

**Figure 4:**
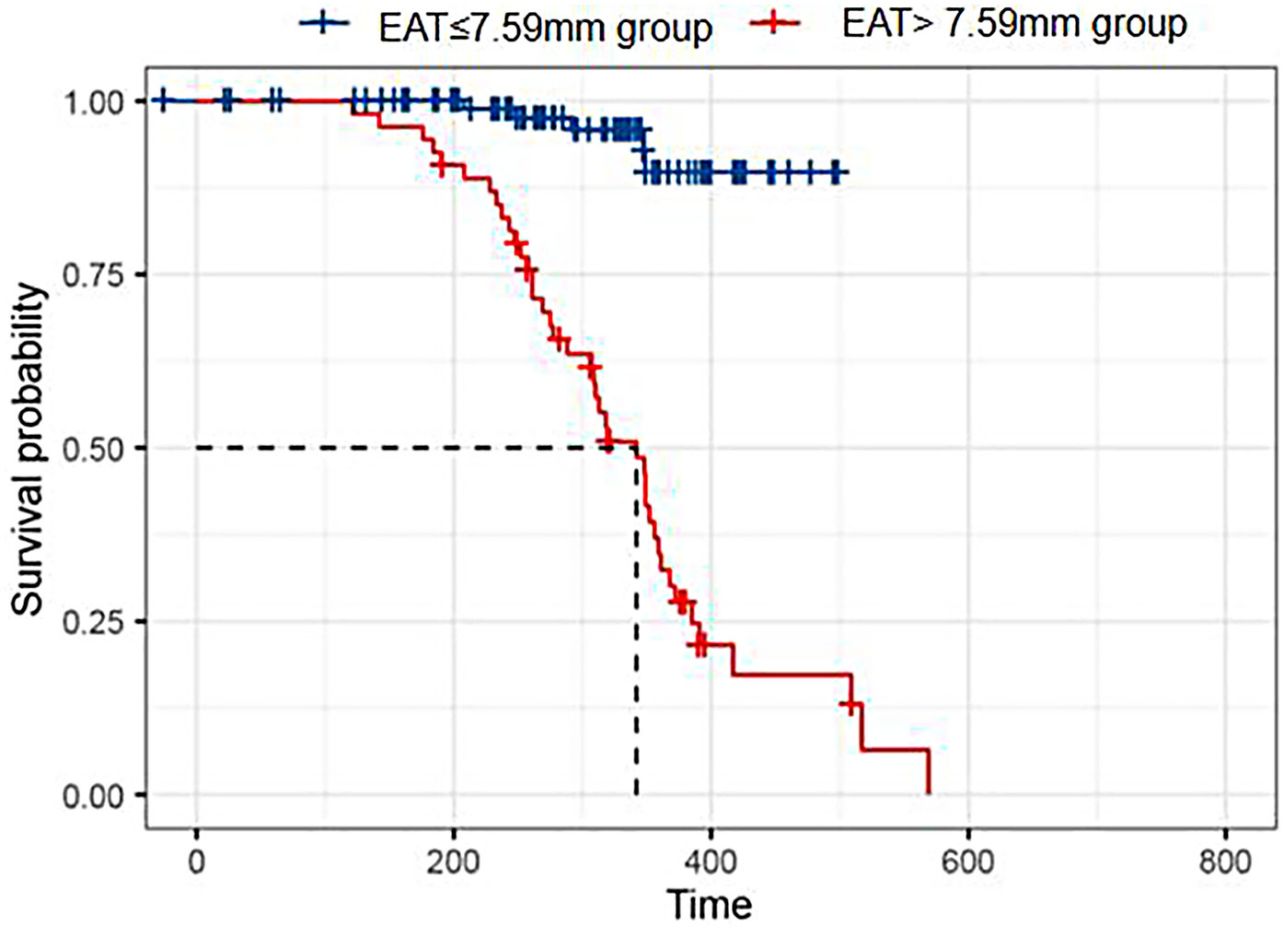
Kaplan–Meier survival analysis. Kaplan–Meier curves comparing event-free survival between HFpEF patients with EAT thickness >7.59 and ≤7.59 mm. The higher EAT group showed significantly reduced survival rates over follow-up.

For Kaplan–Meier analysis, patients were *post hoc* stratified for a higher EAT cutoff of 7.59 mm to demonstrate time-to-event differences. The median follow-up time was 311.50 days (interquartile range 242.75–361.00). Patients with EAT > 7.59 mm had a significantly greater frequency of adverse events compared with patients with EAT ≤ 7.59 mm (*P* < 0.001). The survival curve is compared in Figure 4.

The difference between the ROC-derived cutoff (5.65 mm) from the KM stratification threshold (7.59 mm) is a consequence of the *post hoc* methodology of KM stratification based solely upon the distribution of EAT values in the study sample.

### 3.7 Multifactorial analysis of poor prognosis Cox in HFpEF patients

To achieve model robustness in the context of the 44 events (≥10 events per variable), no more than 4–5 variables could be included in the final multivariate Cox regression model.

Variables based on clinical judgment and univariate *P* < 0.10 included hemoglobin, blood lactate, EAT thickness, LAVI, and presence of hypertension.

The final Cox regression model revealed that low hemoglobin level (HR = 0.850, 95% CI: 0.800–0.903, *P* < 0.001), elevated blood lactate level (HR = 1.452, 95% CI: 1.050–2.008, *P* = 0.024), and elevated EAT thickness (HR = 1.642, 95% CI: 1.153–2.339, *P* = 0.006) remained independent risk factors for a poor prognosis.

LAVI and hypertension were not retained in the final model as a result of multicollinearity or non-statistical significance following adjustment. The updated results can be seen in Table 4.

**Table 4:** Revised multivariate Cox regression analysis of risk factors for poor prognosis in patients with HFpEF.

Factor	*β*	SE	Wald *χ*²	*P*-value	HR (95% CI)
Hemoglobin	−0.162	0.031	17.548	<0.001	0.848 (0.802–0.896)
Blood lactate	0.373	0.165	5.078	0.018	1.441 (1.066–1.948)
EAT Thickness	0.496	0.181	7.534	0.006	1.629 (1.153–2.300)
Scr	0.010	0.009	1.279	0.020	1.048 (1.007–1.091)
eGFR	−0.017	0.009	3.924	0.043	1.891 (1.020–3.505)

EAT, epicardial adipose tissue; LAVI, left atrial volume index. Variables were chosen based on univariate significance, clinical relevance, and collinearity diagnostics. The multivariable model accorded with the ≥10 events-per-variable rule.

### 3.8 Patient flow and follow-up completeness

**Figure 5:**
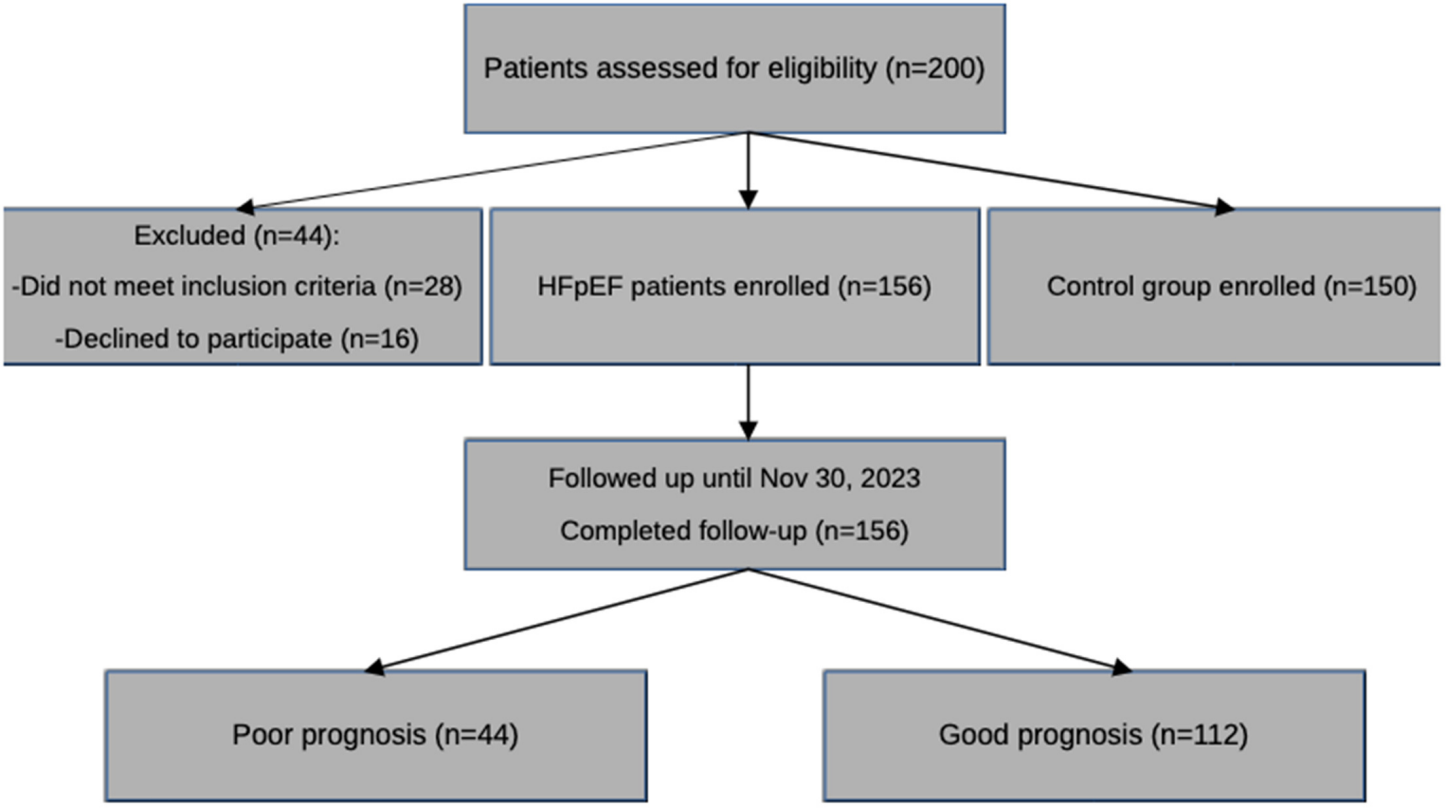
CONSORT-style flow diagram of patient inclusion and follow-up. This flowchart outlines the selection process of study participants, exclusions, and follow-up outcomes, including the number of patients analyzed and those censored during the 1-year follow-up period.

A flow diagram based on CONSORT guidelines was created to summarize the selection, inclusion, and follow-up of patients. All 156 patients completed follow-up, and there were no lost to follow-up or withdrawn consent cases. This fully ensured that data were available and there was no loss to survival analysis (Figure 5).

## 4 Discussion

Heart failure is a clinical syndrome in which the typical signs and symptoms are caused by structural and/or functional cardiac abnormalities, resulting in reduced cardiac output or increased intracardiac pressure at rest or during stress, thereby reducing the return of blood to the cardiac veins. It cannot flow out sufficiently, resulting in venous blood stasis and insufficient blood supply in the blood vessels, which causes a syndrome of cardiac circulatory dysfunction (12–14). Heart failure is the most prominent cause of hospitalization worldwide, affecting 3.6 million new patients annually and causing a socioeconomic burden of billions of dollars annually. Heart failure is the leading cause of hospitalization for people aged 65 and above in China, and the mortality rate is high (15). Although patients with HFpEF have higher ejection fractions, echocardiography shows that patients with HFpEF are similar to patients with heart failure with reduced ejection fraction, and the pathogenesis of HFpEF is more heterogeneous and complex, which is mainly related to ventricular diastolic dysfunction (16, 17). Compared with heart failure patients with reduced ejection fraction, the clinical treatment effect of HFpEF is poor, and the mortality rate is even higher than the former. Therefore, the prognosis of HFpEF patients is a current research hotspot.

Clinically, HFpEF is mainly characterized by diastolic dysfunction, accompanied by left ventricular hypertrophy, left atrium enlargement, decreased diastolic function, impaired cardiac function, inflammatory infiltration of cardiac tissue, and collagen deposition, leading to ventricular remodeling. Left ventricular end-diastolic diameter (LVEDD), left atrial diameter (LAD), LVMI, and LVEF are indicators reflecting ventricular remodeling. The results of this study showed that EAT thickness, LVMI, LAVI, and *E*/*e*′ were higher in the observation group than in the control group (*P* < 0.05), and LVEF was lower than in the control group (*P* < 0.05). It is suggested that echocardiographic parameters can reflect changes in ventricular morphology and configuration in patients with heart failure occurrence and development, which are closely related to changes in the above parameter values. LVEF is routinely used to evaluate the overall systolic function of the left ventricle.

EAT is visceral fat deposited between the visceral pericardium and the myocardium and in direct contact with the myocardium and coronary arteries. From a physiological perspective, EAT acts as a temperature regulator and provides energy to the myocardium. Furthermore, EAT behaves as a metabolically active endocrine organ and secretes bioactive molecules that affect the heart and coronary arteries through paracrine or angiocrimin effects. It can produce proinflammatory adipokines (such as leptin, TNF-*α*, IL-6, and resistin) and downregulate anti-inflammatory adipokines (such as adiponectin and omentin-1) (18). The thickness of EAT is directly proportional to its proinflammatory effect (19). The increase in EAT thickness indicates that chronic inflammation is more intense, and the cardiac load gradually increases. Inflammatory cytokines regulate the phenotype and function of all cardiomyocytes and inhibit the contractile function of cardiomyocytes. They induce inflammatory activation of macrophages, stimulate microvascular inflammation and dysfunction, and promote a matrix-degrading phenotype of fibroblasts, leading to ventricular remodeling. The addition of proinflammatory factors makes myocardial damage more serious and can mediate the apoptosis of myocardial tissue cells. Direct mechanical compression of the myocardium may also be exerted when the EAT thickness increases, leading to a constrictive pericarditis-like condition. Because of the limited flexibility of the pericardium, increased EAT thickness will lead to a reduction in the expansion ability of the wrapped myocardium, leading to diastolic dysfunction and increased cardiac filling pressure (20–22).

In this study, EAT thickness was negatively correlated with LVEF, while it was positively correlated with LVMI, LAVI, and *E*/*e*′ (all *P* < 0.05), highlighting the link to adverse cardiac remodeling. Notably, these results are in keeping with previous findings. Okuno et al. found increased EAT volume correlated with the diastolic strain rate and adverse remodeling in HFpEF (23). Albani et al. confirmed that the inflammatory signaling process facilitated by EAT influences ventricular stiffness and fibrosis, which is a hallmark of HFpEF (24). Parisi et al. confirmed that echocardiographic EAT thickness was able to independently predict arrhythmia, HF progression, and mortality (25).

EAT is also the heart's visceral fat depot. In a healthy state, EAT represents approximately 20% of the heart's weight, with the main visceral fat depots located around the right coronary artery and left anterior descending coronary artery, with some distribution around the atrium, right ventricular free wall, and apical wall (26). A microscopic evaluation of EAT shows a predominance of white adipocytes, which are responsible for energy storage. The cellularity of EAT is greater than that of subcutaneous or other visceral fat depots, associated with less mature adipocytes and more preadipocytes. The visceral fat of EAT not only provides mechanical protection to the heart and peripheral blood vessels but also acts as a unique endocrine organ. Under pathological conditions, it can facilitate the accumulation of EAT and promote the conversion of EAT into a proinflammatory and profibrotic phenotype (27).

The univariate analysis in this study showed that hemoglobin and eGFR values were lower in the poor prognosis group compared with the good prognosis group (*P* < 0.05), and EAT thickness, blood lactate level, and Scr level were all higher in the poor prognosis group (*P* < 0.05). The Kaplan–Meier survival curves revealed that patients with an EAT thickness >7.59 mm had significantly less event-free survival compared with patients with an EAT thickness ≤7.59 mm. Through the multivariate Cox analysis, we found that increased EAT thickness, lower hemoglobin level, and increased lactate level were independent variables predictive of poor prognosis (all *P* < 0.05). We are in agreement with Maimaituxun et al. (28), who found that EAT volume negatively correlated with global longitudinal strain in HF patients, thus supporting the association between EAT and decrements in systolic function.

In addition, prior studies have shown that HFpEF patients have significantly greater total and total ventricular EAT volumes compared with HFrEF patients, even with BMI matching (29). Another study with a cohort of 155 HFpEF patients showed that greater EAT predicted worse outcomes over 24 months of follow-up (30). In a meta-analysis of 1,983 patients from 12 studies of heart failure, it was shown that increased EAT thickness was significantly correlated to increased BNP levels and HF severity (31–34).

This study demonstrated that there could be a strong predictive power of EAT and, in this case, the ROC curve analysis yielded an AUC of 0.892, a sensitivity rate of 89.13%, and a specificity rate of 88.39%. This suggests that echocardiographic EAT thickness could be a valid non-invasive parameter of prognosis in patients with HFpEF.

## 5 Limitations

This study has several limitations. First, it is a single-center study with a small sample size, which can limit external generalizability. Second, there was limited biomarker data (for instance, BNP or NT-proBNP), which were not available for all patients, and many other prognostic biomarkers were not evaluated or collected during the study period. Thirdly, EAT was assessed using echocardiography rather than CT or MRI, which will minimize spatial accuracy and dimensional accuracy. Fourth, we did not consider the factor of interobserver variability in measuring EAT. Finally, the observational nature of this study limits the potential for drawing causal conclusions.

Despite these stated limitations, our findings support the clinical value of EAT thickness as a prognostic marker for HFpEF and suggest a further exploration of how EAT luminosity could be of value for risk refinement and risk stratification. There is a need for future multicenter studies using volumetric imaging modalities to confirm our findings.

## 6 Conclusion

EAT thickness is independently associated with adverse events in patients with HFpEF. EAT thickness may represent a simple, non-invasive, and readily available measure of cardiac remodeling and risk stratification. Additional multicenter studies are necessary to confirm its prognostic utility.

## Data Availability

The raw data supporting the conclusions of this article will be made available by the authors without undue reservation.
